# A protocol for the Development of Core Outcome Sets for Endodontic Treatment modalities (COSET): an international consensus process

**DOI:** 10.1186/s13063-021-05764-x

**Published:** 2021-11-17

**Authors:** I. A. El Karim, H. F. Duncan, S. Cushley, V. Nagendrababu, L. L. Kirkevang, C. Kruse, B. S. Chong, P. K. Shah, M. Lappin, C. McLister, F. T. Lundy, M. Clarke

**Affiliations:** 1 School of Medicine, Dentistry and Biomedical Sciences, Queen’s University Belfast, Belfast, Northern Ireland; 2 Division of Restorative Dentistry & Periodontology, Dublin Dental University Hospital, Trinity College Dublin, Dublin, Ireland; 3 Department of Preventive and Restorative Dentistry, College of Dental Medicine, University of Sharjah, Sharjah, United Arab Emirates; 4 Department of Dentistry and Oral Health, Aarhus University, Aarhus, Denmark; 5 Institute of Dentistry, Barts and The London School of Medicine and Dentistry, Queen Mary University of London, London, UK

**Keywords:** Endodontics, Root canal treatment, Vital pulp treatment, Clinician-centred outcomes, Clinical endpoints, Surgical endodontics, Patient-reported outcomes

## Abstract

**Background:**

The outcome of endodontic treatment is generally assessed using a range of patient and clinician-centred, non-standardised clinical and radiographic outcome measures. This makes it difficult to synthesise evidence for systematic analysis of the literature and the development of clinical guidelines. Core outcome sets (COS) represent a standardised list of outcomes that should be measured and reported in all clinical studies in a particular field. Recently, clinical researchers and guideline developers have focussed on the need for the integration of a patient-reported COS with clinician-centred measures. This study aims to develop a COS that includes both patient-reported outcomes and clinician-centred measures for various endodontic treatment modalities to be used in clinical research and practice.

**Methods:**

To identify reported outcomes (including when and how they are measured), systematic reviews and their included clinical studies, which focus on the outcome of endodontic treatment and were published between 1990 and 2020 will be screened. The COSs will be defined by a consensus process involving key stakeholders using semi-structured interviews and an online Delphi methodology followed by an interactive virtual consensus meeting. A heterogeneous group of key ‘stakeholders’ including patients, general dental practitioners, endodontists, endodontic teachers, clinical researchers, students and policy-makers will be invited to participate. Patients will establish, via interactive interviews, which outcomes they value and feel should be included in a COS. In the Delphi process, other stakeholders will be asked to prioritise outcomes identified from the literature and patient interviews and will have the opportunity at the end of the first round to add outcomes that are not included, but which they consider relevant. Feedback will be provided in the second round, when participants will be asked to prioritise the list again. If consensus is reached, the remaining outcomes will be discussed at an online meeting and agreement established via defined consensus rules of outcome inclusion. If consensus is not reached after the second round, a third round will be conducted with feedback, followed by the online meeting. Following the identification of a COS, we will proceed to identify how and when these outcomes are measured.

**Discussion:**

Using a rigorous methodology, the proposed consensus process aims to develop a COS for endodontic treatment that will be relevant to stakeholders. The results of the study will be shared with participants and COS users. To increase COS uptake, it will also be actively shared with clinical guideline developers, research funders and the editors of general dental and endodontology journals.

**Trial registration:**

COMET 1879. 21 May 2021.

## Introduction

### Background

Endodontic treatment aims to retain teeth, by maintaining pulp vitality and/or health of periapical tissues by preventing and treating infection, and comprises a fundamental part of everyday dental practice. The scope of the treatment includes, but is not limited to, non-surgical and surgical root canal treatment (RCTx) and vital pulp treatment (VPT).

RCTx is usually indicated in teeth in which the dental pulp is severely inflamed (irreversible pulpitis) or when the pulp becomes necrotic with or without infection, but occasionally elective removal of healthy pulp tissue may be indicated for restorative reasons [1]. The treatment involves removing infection primarily from the root canal system, by cleaning, shaping and filling of the root canals. Although complex, the treatment is highly successful when performed to a high standard [2, 3]. The outcome of RCTx is generally assessed by clinical and radiographic examination using a range of criteria. These assessment tools are largely clinician-centred, rather than patient-centred. If primary RCTx fails, teeth can be retreated conventionally or surgically. The outcomes of retreatment are assessed in the same way as primary treatment, with clinical and radiographic examination using various criteria [4]. Some studies reporting outcomes of surgical and non-surgical RCTx use clinical and radiographic criteria individually or as a combined outcome [2, 5]. Others have reported tooth survival or need for further treatment as the outcome [6]. Within the clinical criteria, there is often marked variation in the way clinical outcomes are reported. Radiographic examination is often carried out using conventional imaging, but recently outcomes are also reported using cone beam tomography (CBCT) [7]. There is marked heterogeneity in the reporting of outcomes in published studies and a lack of consistency in reporting the outcomes of randomised controlled trials (RCT).

VPT is a re-emerging minimally invasive endodontic treatment that aims to maintain the vitality of all or part of the dental pulp. There are two main VPT modalities for treating teeth with deep caries and compromised pulp; pulp capping and pulpotomy. Pulp capping (direct or indirect) is a type of VPT generally indicated for teeth with deep caries in which the pulp is not pathologically exposed or is mildly symptomatic [8]. Pulpotomy is indicated for teeth in which the pulp is cariously exposed, and the procedure involves removal of a small part of the inflamed pulp (partial pulpotomy) or amputation of the coronal pulp (complete pulpotomy) depending on the extent of the inflammation and patient symptoms [8]. The outcome of VPT depends on the treatment modality and, as with RCTx, various outcomes including clinical and radiographic outcomes are reported to indicate its success, but with a lack of standardised outcome measures.

A biologically based endodontic therapy known as revascularization or revitalization that aims to promote normal physiological development in immature permanent teeth with pulpal necrosis is an emerging endodontic treatment modality [9]. Like other endodontic treatments, various outcomes are reported for revascularisation procedures that are mainly clinician focussed with no consensus [10].

The absence of standardised outcomes is reflected in the quality of many systematic reviews reporting the effects of endodontic treatment on permanent teeth [2, 11, 12] and has been acknowledged in the European Society of Endodontology (ESE) position statement on the management of deep caries and exposed pulp [8]. For RCTx, the principal outcome reported is periapical health, diagnosed clinically and radiographically. The same also applies to surgical endodontics where healing of periapical tissue is generally regarded as an important outcome. In VPT, maintenance of pulp vitality is considered the primary outcome. There are, however, other outcomes that are often inconsistently reported including tooth survival [6, 13], integrity of restoration, pathological changes (calcification, resorption, etc.) [14] and patient-reported outcomes such as symptoms and oral health-related quality of life measures (OHRQoL).

### Why are core outcome sets important in endodontics?

Core outcome sets (COS) are defined as an agreed standardised set of outcomes that should be measured and reported as a minimum in all trials in a particular field [15]. COS should be registered to support the selection of outcomes and outcome measures in clinical trials, the delivery of routine care [16] and the quality of systematic reviews [17].

The selection of appropriate outcomes is essential if clinical studies are to enable direct comparison between the effects of different interventions with minimal bias. Development and implementation of COS minimise bias in the selection and reporting of outcomes, thereby facilitating evidence synthesis for systematic reviews [15, 18]. Indeed, the bulk of the systematic reviews on the outcome of endodontic treatment reported problems when comparing outcome measures [2, 11, 19]. Outcome reporting bias is likely to affect not only systematic reviews but applies to published research in general, with a resultant negative impact on clinical guideline development and ultimately patient care. The GRADE (Grading of Recommendations Assessment, Development and Evaluation) group, supported by Cochrane [20] and the World Health Organization (WHO) [21] for the development of guideline recommendations, recognises the need to identify a relevant COS for use in the research that will underpin those recommendations.

In dentistry, COS has been or is being developed for various dental treatments including traumatic dental injuries [22], periodontology [23] and orthodontics [24]. Endodontology is an important field in dentistry for which COS are needed. Recently, the ESE developed a focused but limited COS for S3 level clinical practice guidelines [25]. This process was limited to the guideline steering group and was not representative of all stakeholders particularly, service users and patients. There is therefore a need for the development of COS which include full representation of stakeholders so that clinicians, researchers, patients, the general public, policymakers and public health professionals will benefit.

### Aim

The aim of this study is to develop COS suitable for assessing endodontic treatment outcomes in permanent teeth after any form of endodontic treatment including non-surgical and surgical RCTx, VPT and revitalisation procedures.

### Scope

The COS is designed for use in both research and clinical practice, including any healthcare setting in which endodontic treatment for adults with pulpal and periapical disease is carried out.

### Objectives

1. Perform scoping reviews to identify a list of potentially important outcomes from published studies investigating outcomes of various endodontic treatment modalities.
2. Identify a list of potentially important outcomes reported by patients in semi-structured interviews to augment the list generated from the scoping review process.
3. To reach consensus regarding the most important outcomes from the perspective of patients and clinicians by using consensus.
4. To identify appropriate outcome measurement instruments (OMIs) to be used in the reporting of the COS and the appropriate time points at which the outcomes should be measured.

## Methods

**Fig. 1:**
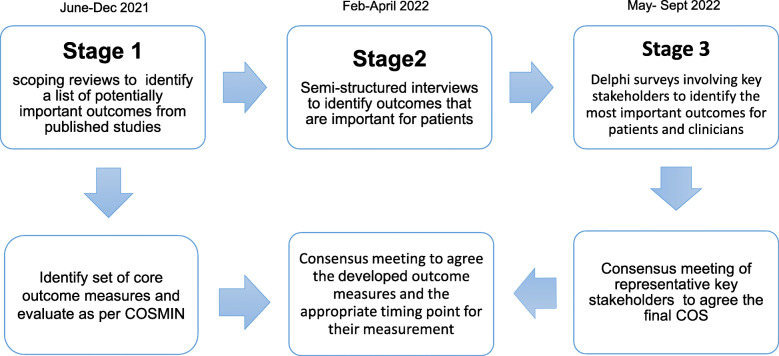
Project outline

This protocol is reported in line with the COS-STAP Statement [26]. The entire methodology followed to develop the Core Outcome Sets for Endodontic Treatment modalities (COSET) is shown in Fig. 1.

### Determine existing outcomes used for endodontic treatment

A scoping review of previous systematic reviews and their included studies will be carried out to identify all outcomes that are reported in endodontic treatment research, including studies evaluating outcomes of different endodontic treatment modalities or prevalence of different types of endodontic treatments. A systematic literature search will be carried out using specific search terms and the following sources: PubMed, MEDLINE, OVID, Embase, Cochrane Database of Systematic Reviews, Web of Science citations and grey literature. Searches will be carried out for reviews published from 1 January 1990 to 31 December 2020. Specific search strategy for each endodontic treatment modality will be developed using appropriate search terms for non-surgical and surgical RCTx, VPT and revitalisation procedures performed in human permanent teeth. Inclusion criteria will be clinical studies in humans involving permanent teeth only and that reported on outcomes of any specific endodontic treatment modality. A minimum of 1-year follow-up and a maximum of as long as possible will be required for reported long term outcomes. Studies not published in the English language or in full text will be excluded. Two independent assessors will carry out study selection and data extraction for each endodontic treatment modality. Data to be collected will include type of intervention, outcomes measured and outcomes reported (clinical, radiographic, patient-reported or any other) and how the outcome is measured and duration of follow-up. A list of outcomes will be generated from these data for use in the subsequent consensus process.

### Patient-reported outcomes

Patients will be recruited to participate in semi-structured qualitative interviews to establish which outcomes they value and report to be the most important conclusion of endodontic treatment. All participants will be adults aged 18 years or older and include those who enrolled in a clinical trial investigating different endodontic treatment modalities and are willing to participate. Purposive sampling will ensure diversity in terms of demographics and type of endodontic treatment experience. This will aim at capturing potential participant differences as previously described for qualitative research [24]. It is planned to recruit and interview the minimum number of participants required to achieve data saturation. The researchers will decide when saturation has been reached.

Semi-structured telephone interviews, utilising open-ended questions will be conducted. Conducting the interviews on a 1:1 basis on the telephone should improve accessibility and maximise participant engagement. The questions will be based on the list of outcomes generated from the literature review with patients being asked to prioritise and rank those outcomes which they feel are the most important and should be included in the COS. Interviews will be audio-recorded, transcribed verbatim and a thematic analysis undertaken.

### Consensus process

#### Stakeholder involvement

If the findings are to influence policy and practice, then the outcomes in the COS need to be relevant and important to key stakeholders. Appropriate key stakeholders include patients, general dental practitioners, specialist endodontists, policy makers, students, endodontic teachers, industry representatives and clinical researchers. These will all be included in the consensus panel with adequate representation from each group. As there is no agreement on appropriate panel size for achieving consensus with the Delphi approach [27], a minimum recruitment of 50 participants will be attempted; however, if saturation of data is not reached with this number, further participants will be recruited. The aim is to recruit a heterogeneous sample, representative of all stakeholders.

Inclusion of patients is critical for the project, as patient contributions help to identify outcomes which have not previously been considered by the other stakeholders [28], and patient-reported outcomes are often ignored in endodontic research. Patient representatives will be identified from those attending their dentist for endodontic treatment. Endodontists, general dental practitioners, students, policy makers and researchers will be identified and contacted through their employers or professional bodies.

A diverse and geographically representative group of senior academics, endodontists and general practitioners will form the steering group for this project. All members of the group will fulfil the following criteria for eligibility: (i) working for at least 5 years in the field of endodontics or another dental science, (ii) have previously published in the area of evidenced-based dentistry, (iii) have a minimum of 10-year academic experience post-qualification and (iv) have no conflict of interest in developing a COS. The members of the group will be invited to participate in the process of COS development as outlined in this document.

#### Definition of the consensus

The first phase in the consensus process is to agree what to measure, i.e. the principal outcome. In the second phase, decisions will be made about how and when the outcome should be measured. The aim is to build solid consensus from a group of diverse users using a rigorous methodology.

#### Delphi consensus

Consensus will be reached using Delphi surveys as previously recommended [18]. This process comprises sequential questionnaires answered anonymously by the participants. The Delphi process has advantages of independent opinion gathering and feasibility for wide geographical representation. An invitation will be sent to potential participants with information on the COS and the consensus process. Those who reply and agree to participate will be invited to contribute to round one of the Delphi surveys. The surveys will be hosted in Google forms and piloted for validation prior to distribution. The aim is to represent all stakeholders to achieve international consensus and an anticipated minimum of 50 participants. The outcomes will be described in lay terms with the help of patient and public involvement (PPI) representatives. Participants will be asked to score each outcome using the scale proposed by the GRADE group, in which 1 to 3 signifies an outcome of relatively little importance, 4 to 6 signifies important but not critical, and 7 to 9 a critical outcome. Participants will also be asked to suggest any outcome measures that they would consider relevant but which were not included in the original list.

For an outcome to qualify for the second round of the Delphi process, ≥70% of responders should score it 7–9 and < 15% should score it 1–3. Participants who complete round 1 will be invited to complete round 2. They will be provided with feedback on the previous round for each outcome and given the opportunity to rescore each of the outcomes remaining in the e-Delphi process, if they wish.

It is expected that three rounds may be required to complete the Delphi process to identify candidate outcomes for the consensus meeting. However, if consensus is clear after two rounds, the third round will not take place. All consensus critical outcomes (rated as 7–9) by ≥70% of responders and < 15% of responders scored the outcome of little importance (rated as 1–3) will be put forward to the COS consensus meeting. To reduce attrition, the researchers will send a reminder via text or email at the beginning of every week.

#### Online consensus meeting

Participants who complete all rounds of the Delphi survey will be invited to attend the online consensus meeting. To ensure effective discussion, up to 20 participants will be chosen at random from those expressing willingness to attend, but ensuring representation from the key stakeholder groups. The meeting will be facilitated with the help of an expert in group consensus meeting methodology and two clinicians.

#### Selection of instruments to measure the core outcomes

Following completion of the identification of COS, we will then identify a set of core outcome measures. The identified outcome measures will be evaluated according to the nine measurement properties identified by the COnsensus-based Standards for the selection of health status Measurement INstruments (COSMIN) initiative, including internal consistency, reliability, measurement error, content validity, structural validity, hypotheses testing, cross-cultural validity, criterion validity and responsiveness [29]. A consensus meeting will also be organised to agree on the developed outcome measures and the appropriate time points for their measurement.

### Research ethics

Ethics approval was obtained from the Queen’s University Belfast Faculty Research Ethics Committee and the Office of Research Ethics Committee N. Ireland. Informed consent will be obtained from all participants.

## Discussion

The development, dissemination and implementation of a COS for endodontic treatment will ensure standardised use of outcomes in research and clinical practice. This study will apply sound methodology and include key stakeholders in the selection of outcomes and their measurement. The addition of patient-reported outcomes is a strength of this proposal and ensures a focus on treatment outcomes that are relevant for patients. The results of this study will be shared with participants and stakeholders including publication in peer-reviewed international journals and conference presentations. To increase uptake, the COS will also be actively shared with clinical guideline developers, research funders, specialist endodontology and other dental journal editors.

### Study status

The study is registered on the COMET website, registration number 1879. The study timeline is provided in Table 1.

**Table 1:** COS-STAP checklist

Title/abstract			Page
**Title**	1a	Identify in the title that the paper describes the protocol for the planned development of a COS	1
**Abstract**	1b	Provide a structured abstract	2–3
**Introduction**			
**Background and objectives**	2a	Describe the background and explain the rationale for developing the COS, and identify the reasons why a COS is needed and the potential barriers to its implementation	4–7
	2b	Describe the specific objectives with reference to developing a COS	8
**Scope**	3a	Describe the health condition(s) and population(s) that will be covered by the COS	8
	3b	Describe the intervention(s) that will be covered by the COS	8
	3c	Describe the context of use for which the COS is to be applied	8
**Methods**			
**Stakeholders**	4	Describe the stakeholder groups to be involved in the COS development process, the nature of and rationale for their involvement and also how the individuals will be identified; this should cover involvement both as members of the research team and as participants in the study	10–11
**Information sources**	5a	Describe the information sources that will be used to identify the list of outcomes. Outline the methods or reference other protocols/papers	9
	5b	Describe how outcomes may be dropped/combined, with reasons	9
**Consensus process**	6	Describe the plans for how the consensus process will be undertaken	11–13
**Consensus definition**	7a	Describe the consensus definition	11
**Analysis**	7b	Describe the procedure for determining how outcomes will be added/combined/dropped from consideration during the consensus process	11–12
**Outcome scoring/feedback**	8	Describe how outcomes will be scored and summarised, describe how participants will receive feedback during the consensus process	12
**Missing data**	9	Describe how missing data will be handled during the consensus process	12
**Ethics and dissemination**			
**Ethics approval/informed consent**	10	Describe any plans for obtaining research ethics committee/institutional review board approval in relation to the consensus process and describe how informed consent will be obtained (if relevant)	13, 15
**Dissemination**	11	Describe any plans to communicate the results to study participants and COS users, inclusive of methods and timing of dissemination	13
**Administrative information**			
**Funders**	12	Describe sources of funding, role of funders	15
**Conflicts of interest**	13	Describe any potential conflicts of interest within the study team and how they will be managed	15

## Data Availability

Not applicable

## Abbreviations

CINAHL: Cumulative Index of Nursing and Allied Health Literature
COMET Initiative: Core Outcome Measures in Effectiveness Clinical Trials
COS: Core outcome set
COSMIN: COnsensus-based Standards for the selection of health Measurement INstruments
EMBASE: Excerpta Medica database
ESE: European Society of Endodontology
GRADE: Grading of Recommendations Assessment, Development and Evaluation
MMSE: Mini-Mental State Examination
OHRQoL: Oral health-related quality of life measures
PPI: Patient and public involvement
RCT: Randomised controlled trial
RCTx: Root canal treatment
VPT: Vital pulp treatment
